# The role of compounded mouthwash with or without acyclovir in managing chemotherapy-induced oral mucositis in cancer patients: a randomized controlled trial

**DOI:** 10.1186/s12903-025-06324-4

**Published:** 2025-06-21

**Authors:** Amira Abdelnasser, Shaimaa El-Ashwah, Salma Elashwah, Mohamed Mabed

**Affiliations:** 1 PharmD, Clinical Pharmacy, Oncology Center, Mansoura University, Mansoura, Egypt; 2 Clinical Haematology, Internal Medicine, Oncology Center, Mansoura University, Mansoura, Egypt; 3 Medical Oncology, Internal Medicine, Oncology Center, Mansoura University, Mansoura, Egypt

**Keywords:** Chemotherapy-induced oral mucositis, HSCT, Compounded mouthwash, Magic mouthwash, Acyclovir

## Abstract

**Background:**

Chemotherapy-induced oral mucositis (CIOM) is a prevalent and debilitating condition observed in cancer patients, especially in those suffering from hematologic malignancies. The present study assessed the efficacy of a compounded mouthwash, both with and without the addition of acyclovir, in the management of CIOM. Although various treatment options exist for this condition, their effectiveness remains limited, underscoring the necessity for innovative approaches to the formulation of compounded mouthwashes for improved management of CIOM.

**Methods:**

A prospective, double-blind, randomized controlled study design with 110 patients with newly diagnosed acute leukemia or who underwent hematopoietic stem cell transplantation. Participants were allocated randomly to either the acyclovir-containing mouthwash group (Arm A) or the control group without acyclovir (Arm B). The incidence, duration, and severity of mucositis were assessed via standardized scale, the World Health Organization (WHO) scale for oral mucositis.

**Results:**

The incidence of mucositis was significantly lower in Arm A (25.5%) compared to Arm B (45.5%), with a *p*-value of 0.028. Furthermore, the duration of mucositis was significantly shorter in Arm A, exhibiting a median duration of 4.5 days, in contrast to a median of 7.5 days observed in Arm B (*p* = 0.01). Grade 3 mucositis was absent in the acyclovir group, whereas there were five cases reported in Arm B (*p* = 0.045). Logistic regression analysis corroborated the protective effect of the acyclovir-containing mouthwash against the development of mucositis, yielding an odds ratio (OR) of 2.444 with a *p*-value of 0.03.

**Conclusions:**

The incorporation of acyclovir into compounded mouthwash significantly reduced the incidence, severity, and duration of oral mucositis in patients undergoing chemotherapy, indicating its potential for expanded clinical application. Further large-scale studies are warranted to validate these findings and to standardize mouthwash formulations for institutional utilization.

**Trial registration:**

This trial was approved by the Institutional Research Board (IRB) under the code R.23.05. 2176.R1. Date: 20 May 2023.

## Concise summary table of key outcomes

**Table:** 

Outcome		Arm A (with Acyclovir)	Arm B (without Acyclovir)	*p*-value
**Incidence of mucositis**		14/55 (25.5%)	25/55 (45.5%)	0.028
**WHO grade of mucositis severity**	Grade 1	11/14 (78.6%)	10/25 (40.0%)	0.045
	Grade 2	3/14 (21.4%)	10/25 (40.0%)	
	Grade 3	0/14 (0.0%)	5/25 (20.0%)	
**Median duration of mucositis (days)**		4.5 (3–13)	7.5 (2–16)	0.01
**Mouthwash arm (B vs A)**	**p**	**OR**	**95% CI**	
	0.03	2.444	1.090- 5.465	

## Introduction

Hematologic malignancies are a major contributor to cancer-related morbidity and mortality worldwide [1, 2]. Despite advances in chemotherapy and radiotherapy, these treatments often lead to adverse effects such as chemotherapy-induced oral mucositis (CIOM) [3], affecting 30–40% of chemotherapy patients, increasing to 60–85% in hematopoietic stem cell transplant (HSCT) recipients and nearly 90% in head and neck cancer patients undergoing chemo-radiotherapy [4]. The severity of CIOM not only compromises patients'quality of life but also, can lead to treatment delays, increased risk of systemic infections, prolonged hospital stays, higher healthcare costs and a higher mortality rate [5, 6].

Several factors contribute to the exacerbation of oral mucositis, including chemotherapy-induced myelosuppression, which increases susceptibility to bacterial, fungal, and viral infections—particularly herpes simplex virus (HSV) [7–9]. Additional risk factor include dehydration, malnutrition, and even mood disorders, all of which may worsen CIOM outcome [10, 11].

Over the past two decades, numerous therapeutic approaches have been explored for CIOM treatment. Despite extensive research, most CIOM treatments remain palliative, offering only partial relief without significantly reducing the incidence or duration of mucositis. Various international guidelines outline recommendations for managing oral mucositis, but evidence supporting the efficacy of many topical treatments remains inconclusive [12].

Among these, topical mouthwashes, commonly referred to as "magic mouthwash," are widely used. However, their effectiveness remains controversial [13, 14] due to a lack of standardized formulations and limited clinical validation. These mouthwashes typically contain a combination of local anaesthetics, anti-inflammatory agents, and antimicrobials [15, 16] to relieve discomfort and create a protective barrier over ulcerated mucosa. However, aside from cryotherapy, few interventions have demonstrated strong evidence-based efficacy in preventing or treating CIOM [12, 17–19].

Reactivation of latent HSV is increasingly recognized as a contributing factor in CIOM pathogenesis, particularly in immunocompromised cancer patients. This viral reactivation exacerbates mucosal injury, delays healing, and increases the risk of secondary infections [20].

Recent research suggests that acyclovir, a well-established antiviral agent, may not only prevent HSV-related lesions but also reduce mucosal inflammation, thereby potentially mitigating CIOM severity [21]. While acyclovir is routinely used to prevent viral reactivation in immunosuppressed patients, its direct role in CIOM management remains underexplored.

Given the high prevalence and severe consequences of CIOM, there is an urgent need to improve treatment strategies and standardized mouthwash formulations. This study aims to evaluate the effectiveness of a compounded mouthwash containing acyclovir, compared to a non-acyclovir formulation, in managing CIOM. We hypothesize that incorporating acyclovir into a multimodal therapeutic approach may not only provide antiviral protection but also reduce mucosal inflammation and ulceration severity, leading to improved patient outcomes.

### Patients and methods

This a prospective, randomized, double-blind trial was approved by the Institutional Research Board (IRB) under the code R.23.05.2176.R1. Clinical and laboratory data were collected from patients treated at oncology center, Mansoura University, Egypt. Eligible participants included newly diagnosed acute leukemia patients receiving intensive chemotherapy or undergoing HSCT. Eligible patients were required to be over 18 years old and able to communicate verbally or in writing. Patients were excluded if they had an Eastern Cooperative Oncology Group (ECOG) performance score ≥ 2, were on investigational agents, (e.g., viscous lidocaine), or had known allergies to study components.

### Sample size

The sample size was determined using an online calculator (https://clincalc.com/stats/samplesize.aspx), based on the incidence of CIOM in patients receiving acyclovir prophylaxis (study group) versus those who did not (control group) [22]. The calculation was performed with a 5% alpha error and a study power of 95%, resulting in a minimum requirement of 41 participants per group. To account for potential dropouts, the initial target enrolment was set at 50 subjects per group.

### Recruitment and randomization

**Fig. 1:**
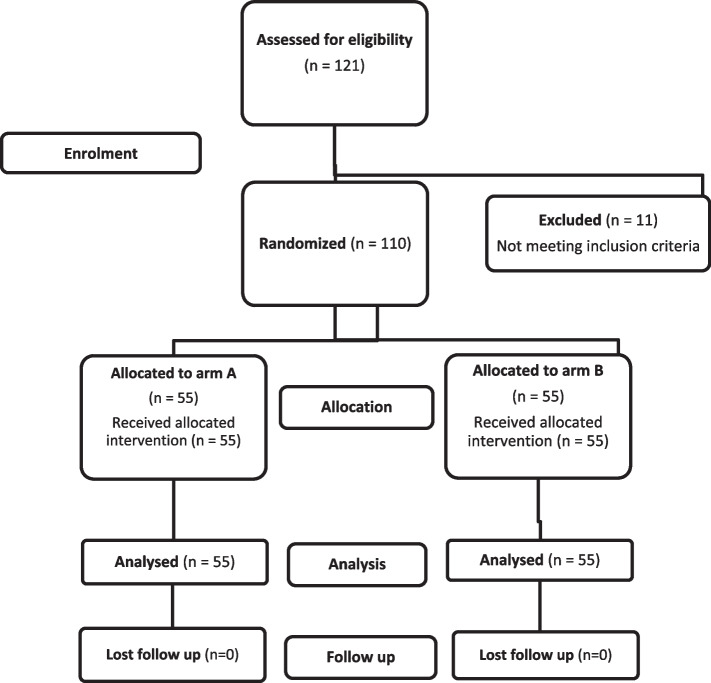
CONSORT flowchart of the study

A total of 121 patients were recruited for the study; 11 patients were excluded because of failure to meet the inclusion criteria. A total of 110 patients completed the study and were randomized to one of two groups, one with a mouthwash with acyclovir arm or one with a mouthwash without acyclovir, using a permuted block randomization method with a 1:1 allocation ratio. The randomization process was conducted in fixed block sizes to ensure equal distribution between the intervention and control groups. To maintain allocation concealment, the randomization sequence was prepared and managed by an independent third party not involved in the study. A flow chart of the study is illustrated in Fig. 1.

The mouthwash formulations for both groups were specifically compounded for this study by sequentially adding each individual component, including acyclovir, to 250 ml of normal saline. No commercially available or pre-prepared mouthwash bases were used. This approach ensured that all ingredients were precisely measured and mixed according to the study protocol. The mouthwash formulation for both groups is detailed in Table 1.

**Table 1:** Compounded mouthwash components

Arm A		Arm B	
**Compounded mouthwash components are:**	**Final Concentration to250 ml of normal saline (mg/ml)**	**Compounded mouthwash components are:**	**Final Concentration to250 ml of normal saline (mg/ml)**
60 mg (0.15%w/v) of Benzydamin (tantum)	0.24	60 mg (0.15%w/v) of Benzydamin (tantum)	0.24
700 mg of AL(OH)3 (Maalox)	2.8	700 mg of AL(OH)3 (Maalox)	2.8
200 mg of nystatin	0.8	200 mg of nystatin	0.8
16 mg of dexamethasone	0.064	16 mg of dexamethasone	0.064
91 mg of diphenhydramine	0.364	91 mg of diphenhydramine	0.364
800 mg of acyclovir	3.2		

As no specific published stability data were available for the compounded mouthwash, the beyond-use date (BUD) was determined according to United States Pharmacopeia (USP 795) and the National Association of Pharmacy Regulatory Authorities (NAPRA) guidelines for non-sterile, water-based mucosal preparations. While USP 795 permits a BUD of up to 30 days at controlled room temperature in the absence of stability data, the formulation was refrigerated (2–8 °C) and used within seven days to ensure optimal integrity and minimize microbial risk. Although no formal stability tests (e.g., pH or microbial load) were conducted, all storage and labelling practices adhered to compounding standards to safeguard product quality and patient safety [23].

Patients were instructed to rinse with 10 ml of the mouthwash for 2–5 min, then spit it out. This was repeated four times daily. For chemotherapy patients, starting one day before chemotherapy initiation and continuing for two weeks. For HSCT patients, starting one day before the conditioning regimen and continuing until discharge.

### Follow-up data

Patients were assessed daily for symptoms, side effects, and mucositis severity and then monitored weekly for two weeks. Follow-up data included the incidence, grade, duration of mucositis, associated gastrointestinal symptoms, antibiotic use and complete blood count at mucositis onset and resolution.

Assessments were performed using the patient-reported oral mucositis scale (PROMS) [24], which was translated into Arabic. The Arabic version was validated through a back-translation method and pre-tested on a subset of patients for comprehensibility and reliability. Assessments were conducted either in person or by phone to ensure comprehensive follow-up.

The effectiveness of the compounded mouthwash was assessed based on its ability to prevent CIOM, reduce its severity and duration. The evaluation of oral mucositis was conducted by independent assessors who were blinded to the treatment groups. These assessors performed oral examinations and graded mucositis severity using the WHO scale for oral mucositis [25] without knowledge of the patient's treatment arm. To ensure consistency and reliability in scoring, all assessors underwent standardized training before the study, which included, detailed instruction on the WHO grading criteria.

### Statistical analyses

Data were analyzed using IBM SPSS Statistics for Windows, Version 25.0 (IBM Corp., Armonk, NY, USA). Qualitative variables were compared using the Chi-square test or Fisher’s exact test, while the normality of quantitative variables was assessed using the Kolmogorov–Smirnov test. For normally distributed data, results were presented as mean ± standard deviation (SD) and analyzed using the paired sample t-test. Non-normally distributed data were expressed as median and range and analyzed using the Mann–Whitney U test for independent groups or the Wilcoxon signed-rank test for paired data.

To identify independent factors associated with the disease, logistic regression analysis was performed. A *p*-value of less than 0.05 was considered statistically significant in all analyses.

## Results

A total of 110 patients diagnosed with acute leukemia or who had undergone HSCT following intensive chemotherapy were included in the study. The cohort was composed of 62 males and 48 females, with a median age of 38.5 years (range: 18–66 years). Diagnoses within the cohort included Acute Myeloid Leukemia (AML, 40.0%), Acute Lymphoblastic Leukemia (ALL, 23.6%), Multiple Myeloma (MM, 18.2%), Hodgkin Lymphoma (HL, 10.0%), and Non-Hodgkin Lymphoma (NHL, 8.2%).

In terms of treatment modalities, 54.5% of patients were administered chemotherapy, 36.4% underwent autologous HSCT, and 9.1% received allogeneic HSCT. At baseline, 54.5% of patients exhibited active disease, 43.6% were in complete remission (CR), and 1.8% were categorized as having very good partial remission (VGPR). Participants were randomized evenly into two groups: Arm A, which received an acyclovir-containing mouthwash, and Arm B, which received a mouthwash devoid of acyclovir.

**Fig. 2:**
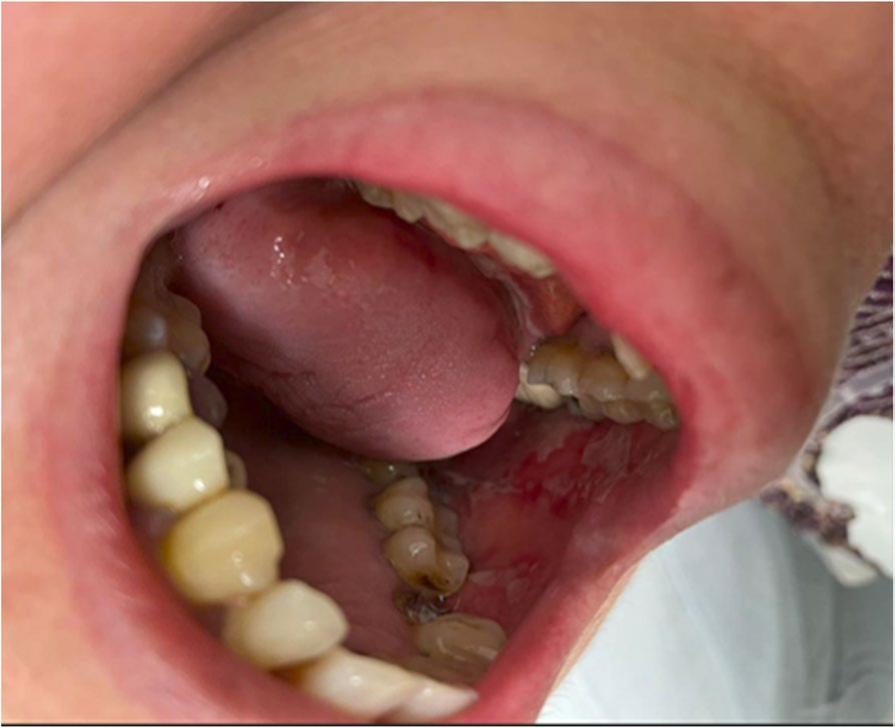
Example of Grade 2 mucositis as scored using WHO oral mucositis criteria

**Fig. 3:**
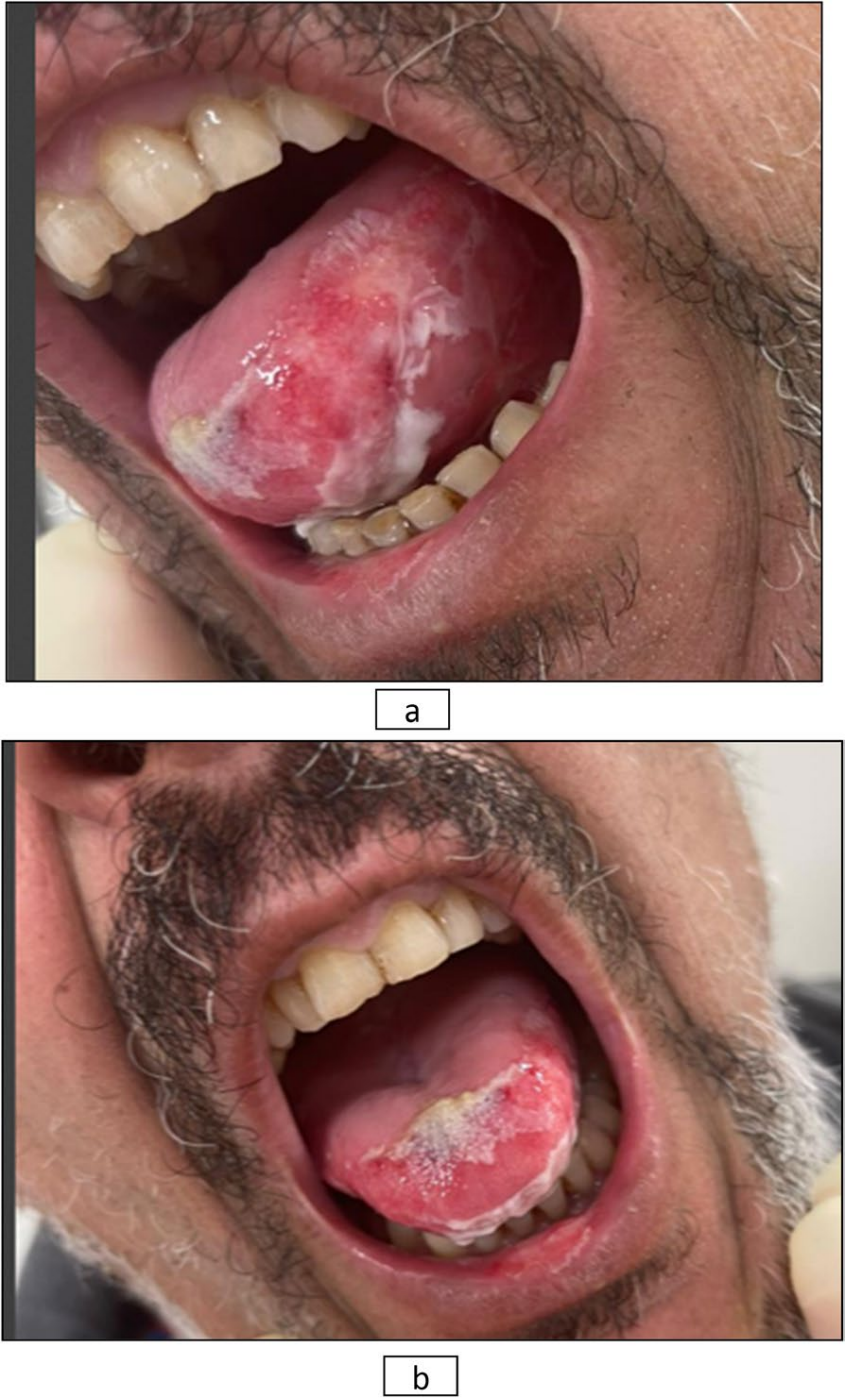
**A**, **B** Example of Grade 3 mucositis as scored using WHO oral mucositis criteria

**Fig. 4:**
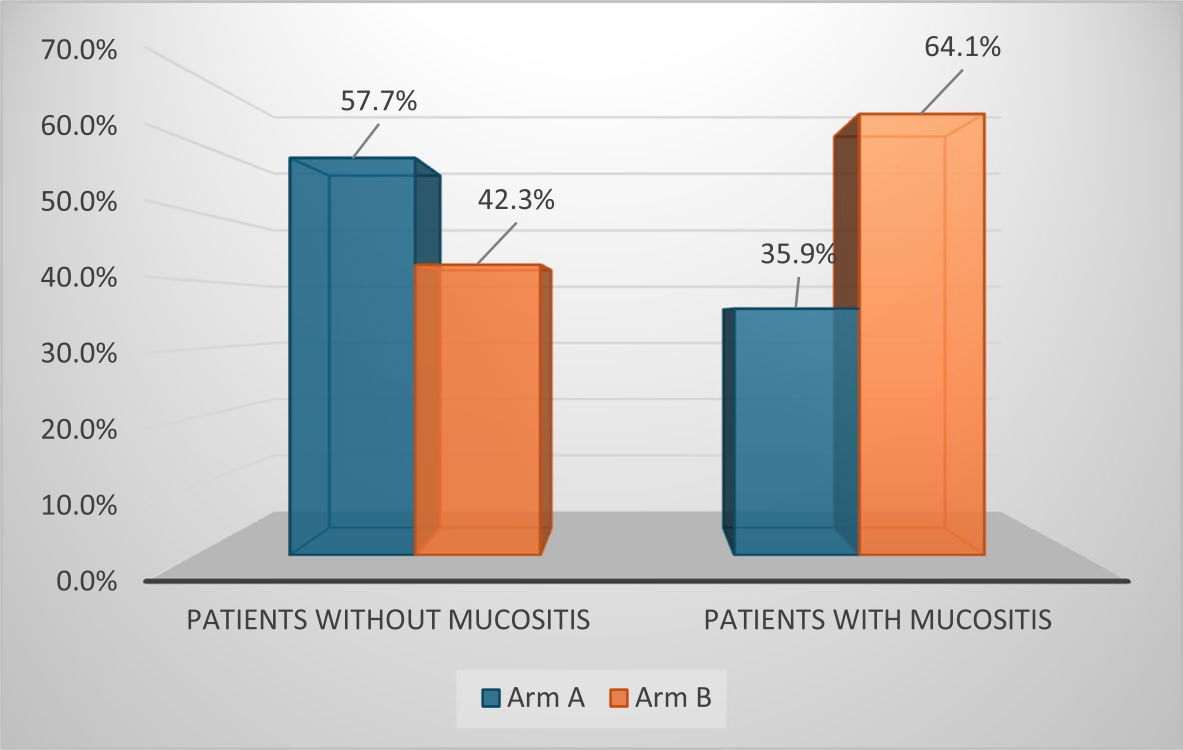
Comparison of incidence of oral mucositis between Arm A and Arm B

**Fig. 5:**
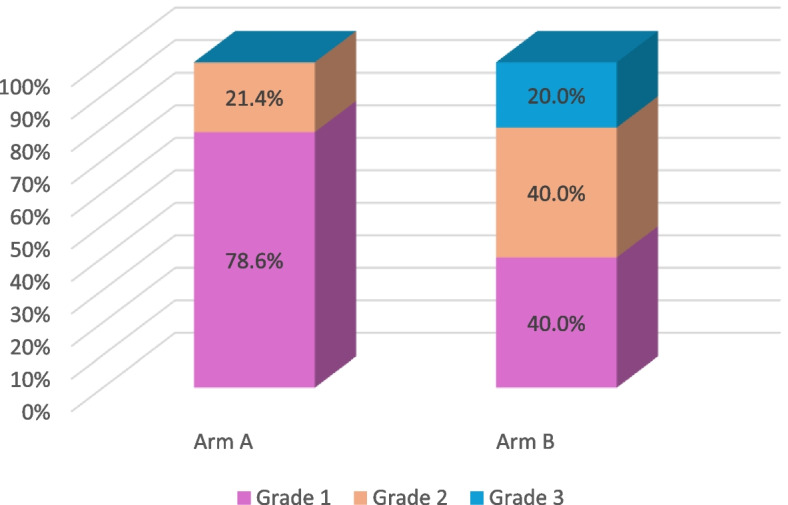
Distribution of oral mucositis severity among study participants

CIOM was observed in 39 patients, representing 35.5% of the total cohort. Among these cases, 53.9% were classified as grade 1, 33.3% as grade 2, and 12.8% as grade 3 CIOM (Figs. 2 and 3a, b). Patients assigned to Arm B exhibited a significantly higher prevalence of CIOM as well as grade 3 CIOM in comparison to those in Arm A, with *p*-values of 0.028 and 0.045, respectively (Table 2; Figs. 4 and 5).

**Table 2:** Patient characteristics among the studied groups

Parameters		Total (*N* = 110)	Arm A (*N* = 55)	Arm B (*N* = 55)	*P* value
Age* (years)	Median (Min–Max)	38.5 (18–66)	37.0 (18–66)	40.0 (18–64)	0.669
**Sex** **(No, (%))**	Male	62 (56.4%)	31 (56.4%)	31 (56.4%)	1.00
	Female	48 (43.6%)	24 (43.6%)	24 (43.6%)	
**Diagnosis** **(No, (%))**	ALL	26 (23.6%)	14 (25.5%)	12 (21.8%)	0.258
	AML	44 (40.0%)	21 (38.2%)	23 (41.8%)	
	HL	11 (10.0%)	8 (14.5%)	3 (5.5%)	
	NHL	9 (8.2%)	2 (3.6%)	7 (12.7%)	
	MM	20 (18.2%)	10 (18.2%)	10 (18.2%)	
**Method of treatment** **(No, (%))**	Allo HSCT	10 (9.1%)	5 (9.1%)	5 (9.1%)	1.00
	Auto HSCT	40 (36.4%)	20 (36.4%)	20 (34.6%)	
	Chemotherapy	60 (54.5%)	30 (54.5%)	30 (54.5%)	
**Type of chemotherapy** **(No, (%))**	7+3	40 (36.3%)	20 (36.4%)	20 (36.4%)	1.00
	Hyper CVAD	20 (18.2%)	10 (18.2%)	10 (18.2%)	
	BU-CY	10 (9.1%)	5 (9.1%)	5 (9.1%)	
	BeEAM	20 (18.2%)	10 (18.2%)	10 (18.2%)	
	High dose melphalan	20 (18.2%)	10 (18.2%)	10 (18.2%)	
**Status of malignancy** **(No, (%))**	CR ≥ 1	48 (43.6%)	25 (45.5%)	23 (41.8%)	0.353
	VGPR	2 (1.8%)	0 (0.0%)	2 (3.6%)	
	Active	60 (54.5%)	30 (54.5%)	30 (54.5%)	
**Mucositis** **(No, (%))**	Absent	71 (64.5%)	41 (74.5%)	30 (54.5%)	0.028
	Present	39 (35.5%)	14 (25.5%)	25 (45.5%)	
**Grade of mucositis (WHO scale) (No, (%))**	Grade 1	21 (53.9%)	11 (78.6%)	10 (40.0%)	0.045
	Grade 2	13 (33.3%)	3 (21.4%)	10 (40.0%)	
	Grade 3	5 (12.8%)	0 (0.0%)	5 (20.0%)	
Duration of mucositis* (Days)	Median (Min–Max)	5.0 (2–16)	4.5 (3–13)	7.5 (2–16)	0.01
**Neutrophil** at onset of mucositis * (× 10^**9**^**/L)**	Median (Min–Max)	0.133 (0.001–3.048)	0.035 (0.002–3.048)	0.160 (0.001–2.98)	0.239
**Neutrophil** At resolution of mucositis* (× 10^**9**^**/L)**	Median (Min–Max)	0.107 (0.001–14.8)	0.024 (0.001–3.55)	0.112 (0.003–14.8)	0.273
**Associated GIT symptoms** **(No, (%))**	Diarrhea	15 (13.6%)	6 (54.5%)	9 (69.2%)	0.459
	Constipation	7 (6.36%)	3 (27.3%)	4 (30.8%)	
	Abdominal colic	1 (0.9%)	1 (9.1%)	0 (0.0%)	
	GERD	1 (0.9%)	1 (9.1%)	0 (0.0%)	
**Outcome**	Alive	69 (62.7%)	35 (63.6%)	34 (61.8%)	0.844
	Dead	41 (37.3%)	20 (36.4%)	21 (38.2%)	

*ALL *acute lymphoblastic leukemia, *AML *acute myeloid leukemia, *MM* multiple myeloma, *NHL* non-Hodgkin lymphoma, *HD* Hodgkin lymphoma, *Allo-HSCT* allogenic hematopoietic stem cell transplant, *Auto-HSCT* autologous hematopoietic stem cell transplant, *BeEAM* bendamustine, cytarabine, etoposide, melphalan, *BU-CY* busulfan-cyclophosphamide, *CR* complete remission, *VGPR* very good partial response. Continuous variables are expressed as the median (min–max) *. The data were analysed with Mann‒Whitney tests*. The chi-square test was used for comparisons of categorical parameters. *P* values between both groups.

In a comparative analysis of patients with and without mucositis, those with AML demonstrated a higher incidence of CIOM; however, these association did not reach statistical significance (Table 3).

**Table 3:** Comparison of the clinicopathological characteristics as regard patients with and without mucositis

Parameters		Patients without mucositis (*N* = 71)	Patients with mucositis (*N* = 39)	*P*-value
Age* (years)	**Median (Min–Max)**	39.0 (18–66)	37.0 (18–64)	0.755
**Sex** (No, (%))	**Male**	39 (54.9%)	23 (59.0%)	0.682
	**Female**	32 (45.1%)	16 (41.0%)	
**Diagnosis** (No, (%))	**ALL**	19 (26.8%)	7 (19.7%)	0.053
	**AML**	21 (29.6%)	23 (59.0%)	
	**HL**	9 (12.7%)	2 (5.1%)	
	**NHL**	7 (9.9%)	2 (5.1%)	
	**MM**	15 (21.1%)	5 (12.8%)	
**Method of treatment** (No, (%))	**Allo HSCT**	6 (8.5%)	4 (10.3%)	0.098
	**Auto HSCT**	31 (43.7%)	9 (23.1%)	
	**Chemotherapy**	34 (47.9%)	26 (66.7%)	
**Type of chemotherapy** (No, (%))	**7+3**	20 (28.2%)	20 (51.3%)	0.131
	**Hyper CVAD**	14 (19.7%)	6 (15.4%)	
	**BU-CY**	6 (8.5%)	4 (10.34%)	
	**BeEAM**	16 (22.5%)	4 (10.3%)	
	**High dose melphalan**	15 (21.1%)	5 (12.8%)	
**Status of malignancy** (No, (%))	**CR ≥ 1**	35 (49.3%)	13 (33.3%)	0.123
	**VGPR**	2 (2.8%)	0 (0.0%)	
	**Active**	34 (47.9%)	26 (66.7%)	
**Outcome** (No, (%))	**Alive**	48 (67.6%)	21 (53.8%)	0.153
	**Dead**	23 (32.4%)	18 (46.2%)	

Continuous variables are expressed median (min–max)*. Data are Mann–whitney tests*. Chi square test for comparison of categorical parameters. P between both groups

A univariate logistic regression analysis was conducted to identify independent predictors of CIOM. The covariates included in the control group were age, sex, therapeutic modality, malignancy status, and medical arm. The use of mouthwash without acyclovir (Arm B) was found to significantly increase the odds of developing mucositis, with an odds ratio (OR) of 2.444 and a 95% confidence interval (CI) ranging from 1.090 to 5.465 (*p* = 0.03) (Table 4). Clinically, these results indicate that patients utilizing mouthwash without acyclovir were more than twice as likely to develop CIOM compared to those using mouthwash that contained acyclovir. These findings emphasize the protective effect of topical acyclovir in mitigating chemotherapy-induced mucosal damage.

**Table 4:** Univariate analysis to predict the presence of mucositis in patients who did not have mucositis

	p	OR	95% CI	
**Age (years)**	0.789	0.996	0.969	1.024
**Sex**	0.683	0.848	0.384	1.870
**Method of treatment**				
**- Auto HSCT VS allo HSCT**	0.267	0.435	0.100	1.888
**- Chemotherapy VS allo HSCT**	0.844	1.147	0.293	4.488
**Status of malignancy**				
**-Active VS CR**	0.083	2.059	0.910	4.656
**Mouthwash arm (B vs A)**	0.03	2.444	1.090	5.465

Logistic regression analysis for the prediction of patients with mucositis from patients without mucositis. OR, odds ratio; CI, confidence interval; logistic regression was used

Subgroup analysis of the autologous HSCT cohort revealed a lower incidence of CIOM in Arm A compared to Arm B; however, this difference did not reach statistical significance (*p* = 0.451). Male sex was also significantly affected among patients with CIOM (*p* = 0.027). There were no significant differences observed concerning age, diagnosis, or malignancy status within this subgroup (Table 5).

**Table 5:** Comparison of the clinicopathological characteristics for patients underwent Autologous stem cell transplant with and without mucositis

Parameters for auto HSCT (n = 40)		Patients without mucositis (*N* = 31)	Patients with mucositis (*N* = 9)	*P*-value
Age* (years)	**Median (Min–Max)**	49.0 (21–66)	50.0 (21–64)	0.633
**Sex** (No, (%))	**Male**	14 (45.2%)	8 (88.9%)	0.027
	**Female**	17 (54.8%)	1 (11.1%)	
**Diagnosis** (No, (%))	**HL**	9 (29.0%)	2 (22.2%)	0.910
	**NHL**	7 (22.6%)	2 (22.2%)	
	**MM**	15 (48.4%)	5 (55.6%)	
**Status of malignancy** (No, (%))	**CR**	29 (93.5%)	9 (100.0%)	1.00
	**VGPR**	2 (6.5%)	0 (0.0%)	
**Mouth wash** (No, (%))	**Arm A**	17 (54.8%)	3 (33.3%)	0.451
	**Arm B**	14 (45.2%)	6 (66.7%)	
**Outcome** (No, (%))	**Alive**	31 (100.0%)	8 (88.9%)	0.225
	**Dead**	0 (0.0%)	1 (11.1%)	

Continuous variables are expressed median (min–max)*. Data are Mann–whitney tests*. Chi square test for comparison of categorical parameters. P between both groups

Among chemotherapy recipients, the incidence of CIOM was significantly higher in Arm B than in Arm A (*p* = 0.037). Other variables within this subgroup did not demonstrate statistically significant differences (Table 6).

**Table 6:** Comparison of the clinicopathological characteristics for patients receiving chemotherapy with and without mucositis

Parameters for chemotherapy (*n* = 60)		Patients without mucositis (*N* = 34)	Patients with mucositis (*N* = 26)	*P*-value
Age* (years)	**Median (Min–Max)**	36.0 (18–63)	40.5 (18–61)	0.218
**Sex** (No, (%))	**Male**	19 (55.9%)	13 (50.0%)	0.651
	**Female**	15 (44.1%)	13 (50.0%)	
**Diagnosis** (No, (%))	**ALL**	14 (41.2%)	6 (23.1%)	0.141
	**AML**	20 (58.8%)	20 (76.9%)	
**Mouth wash** (No, (%))	**Arm A**	21 (61.8%)	9 (34.6%)	0.037
	**Arm B**	13 (38.2%)	17 (65.4%)	
**Outcome** (No, (%))	**Alive**	12 (35.3%)	9 (34.6%)	1.00
	**Dead**	22 (64.7%)	17 (65.4%)	

Continuous variables are expressed median (min–max)*. Data are Mann–whitney tests*. Chi square test for comparison of categorical parameters. P between both groups

## Discussion

Research conducted in the 1990 s predominantly dismissed oral HSV reactivation as a significant factor in CIOM [26–29]. However, more recent findings have established HSV-1 reactivation as an independent risk factor for the development of CIOM in patients with hematological malignancies (HM) undergoing chemotherapy or HSCT [20, 30–36]. HSV-1 may worsen mucosal injury through mechanisms involving both localized inflammation and direct cytopathic effects, thereby providing a rationale for antiviral treatments such as topical acyclovir [22].

This study enhances the current understanding of the topic by illustrating that patients administered a compounded mouthwash containing acyclovir exhibited a markedly lower incidence and severity of CIOM when compared to those receiving the non-acyclovir formulation. Notably, none of the patients in the acyclovir group experienced grade 3 mucositis, and a higher proportion of these patients presented with only mild (grade 1) lesions. These results indicate that topical acyclovir may confer mucosal protection through its local antiviral activities and potential anti-inflammatory properties [21].

Numerous earlier studies, which primarily focused on the incidence CIOM, have reported inconsistent findings. In contrast, our study evaluated both the incidence and the duration of CIOM symptoms [21, 37–52]. Our results demonstrated a lower incidence and faster resolution of mucositis in the acyclovir treatment group, highlighting a dual benefit in both prevention and symptom management. This differentiation is of clinical significance, as the reduction in both the incidence and duration of mucositis can enhance patients'quality of life, decrease hospital admissions, and lower the risk of complications.

The influence of patient-related factors such as age and sex on the incidence of CIOM has been extensively studied, with inconclusive results. Some research suggests that older adults may be at higher risk due to reduced mucosal regeneration and altered drug metabolism, while paediatric patients may be more vulnerable due to immature mucosal defenses [53–55]. However, consistent with findings from Merlano et al., [56] our data showed no significant association between age and mucositis severity (*p* = 0.669).

Similarly, although some studies have identified female sex as a predictor of CIOM, potentially due to hormonal and pharmacokinetic factors [57, 58], we found no sex-based differences in mucositis severity (*p* = 1.00).

Neutropenia has often been cited as a key contributor to CIOM due to its role in impairing tissue healing and increasing vulnerability to infection [59–61], However, our study found no significant association between neutropenia and CIOM at either onset or resolution, potentially due to variability in chemotherapy regimens or supportive care practices.

Several studies investigating the efficacy of prophylactic agents for CIOM have reported mixed results [21, 37–51]. For instance, topical agents such as benzydamine and chlorhexidine have shown variable efficacy in different patient populations [62]. Topical acyclovir, by targeting viral reactivation, may offer a more consistent benefit in susceptible patients. This highlights the importance of conducting head-to-head trials to compare different mouthwash formulations and optimize CIOM prevention strategies.

A study conducted by Hong et al [22]. investigated the use of systemic acyclovir in reducing CIOM in patients undergoing autologous stem cell transplant, and their findings showed a statistically significant reduction in CIOM cases in the acyclovir group (16.0%) compared to the control group (58.6%) (*P* = 0.001), and also all incidences of oral mucositis in the acyclovir arm were grade 1, highlighting the effectiveness of systemic acyclovir in managing mucositis. Despite routine use of systemic acyclovir at our institution to prevent viral reactivation in stem cell transplant setting, CIOM continues to occur at notable rates. Our findings suggest that even with systemic antiviral prophylaxis, topical delivery may provide added benefit by achieving higher local drug concentrations. Although the observed reduction in mucositis incidence with topical acyclovir in our study did not reach statistical significance (*p* = 0.451), the trend warrants further exploration in larger studies.

Despite its notable strengths, this study is not without limitations. The single-center design and modest sample size restrict the generalizability of our findings, highlighting the necessity for multi-center trials aimed at validating the efficacy of acyclovir-containing mouthwashes across a more diverse patient population. Furthermore, although we followed established compounding guidelines (NAPRA, USP 795), the lack of stability data for the acyclovir-containing formulation raises concerns. Future research should implement standardized formulations accompanied by formal stability testing to ensure both long-term efficacy and safety. Finally, while we made efforts to control for potential confounding variables, unmeasured factors, such as variations in chemotherapy regimens or additional supportive treatments, may have influenced the results.

## Conclusion

The incorporation of acyclovir into compounded mouthwash formulations is associated with a significant reduction in the incidence, severity, and duration of CIOM, indicating its potential for wider clinical applications. To validate these results and establish standardized mouthwash formulations for use in healthcare institutions, further large-scale studies are necessary.

## Data Availability

The datasets used and/or analysed during the current study are available from the corresponding author upon reasonable request.

## Abbreviations

CIOM: Chemotherapy-induced oral mucositis
ECOG: Eastern Cooperative Oncology Group
HSCT: Hematopoietic stem cell transplantation
IRB: Institutional Research Board
NAPRA: National Association of Pharmacy Regulatory Authorities
OMAS: Oral Mucositis Assessment Scale
PROMS: Patient-reported oral mucositis scale
